# Nesfatin-1 alleviated lipopolysaccharide-induced acute lung injury through regulating inflammatory response associated with macrophages modulation

**DOI:** 10.1186/s13019-022-01952-1

**Published:** 2022-08-26

**Authors:** Hongbing Cheng, Yanfang Zhu, Liangji Chen, Yalan Wang

**Affiliations:** Xiantao First People’s Hospital Affiliated to Yangtze University, No. 29, Middle Section of Mianzhou Avenue, Nancheng New District, Xiantao City, 433099 Hubei Province China

**Keywords:** Acute lung injury (ALI), Lung inflammation, Macrophages, Nesfatin-1, p38 MAPK/c-Jun pathway, NF-κB pathway

## Abstract

Acute lung injury (ALI) is a continuum of lung changes associated with uncontrolled excessive lung inflammation. However, the pathogenesis of ALI is still complicated and effective clinical pharmacological management is required. Various signaling pathways are involved in the inflammatory responses of ALI. Here, we aimed to explore the role of nesfatin-1, an amino-acid peptide with anti-inflammatory action, in an LPS-induced ALI mice model, and its role in regulating macrophages in response to LPS stimulation in vitro. This was to clarify the underlying mechanisms of regulating the inflammatory response in the development of ALI. The results show that nesfatin-1 expression was downregulated in the lung tissues of ALI mice compared to control mice. Nesfatin-1 treatment ameliorated the inflammatory response and lung tissue damage in LPS-induced ALI in mice. In vitro studies showed that nesfatin-1 attenuated the generation and release of proinflammatory cytokines interleukin-6 (IL-6), interleukin-1β (IL-1β), and tumor necrosis factor-α (TNF-α) in LPS-induced RAW 264.7 cells. Nesfatin-1 also inhibited reactive oxygen species production and improved superoxide dismutase (SOD) activity in LPS-induced RAW 264.7 cells. These findings suggest that nesfatin-1 exerted a crucial role in regulating the LPS-mediated activation of M1 macrophages. Further mechanism investigations indicated that nesfatin-1 inhibited the activation of p38 MAPK/c-Jun and NF-κB pathways in LPS-induced RAW 264.7 cells, as evidenced by decreased expression levels of p-p38, p-c-Fos, and p-p65. Overall, nesfatin-1 alleviated LPS-induced ALI, which might be attributed to regulating inflammatory response through macrophages modulation.

## Introduction

Acute lung injury (ALI) is characterized by a series of changes in lung tissue including surfactant dysfunction, neutrophil-derived inflammation, lung edema formation, and diffuse alveolar injury [1]. ALI arises from a wide variety of pathological conditions, such as near-drowning, aspiration of gastric contents, trauma, and sepsis [1]. Clinically, ALI frequently results in bilateral pulmonary infiltrates, severe hypoxemia, and lung compliance, resulting insignificant morbidity and often death [2].

Studies on the pathogenesis of ALI have shown that bacterial antigens trigger an inflammatory response that depends on the activation of several pathways [3, 4]. For instance, lipopolysaccharide (LPS) binds to Toll-like receptor (TLR) 4 and leads to the activation of intracellular kinases, thereby activating the proinflammatory pathways [5]. These signals converge in the regulation of diverse transcription factors that induce the synthesis of pro-inflammatory molecules mediating the molecular pathophysiology of inflammatory responses. As a result, neutrophils are recruited from the circulation, alveolocapillary permeability increases, and pneumocytes are yielded to cell death, all of which result in further injury [5]. Consequently, it is hypothesized that the regulation of inflammation is correlated with improved outcomes of ALI.

Nesfatin-1 is an amino-acid peptide, which is widely found in digestive, nervous, and adipose tissues [6]. It is evident that nesfatin-1 has multiple functions, including modulation of inflammation, stress, and blood pressure, anorexigenic, emotion, cancer, metabolism, and reproduction [7–9]. Particularly, nesfatin-1 was found to possess protective action on lung injury [10]. Serum levels of nesfatin-1 in lung cancer patients are lower than those in healthy subjects, which are also associated with weight loss in lung cancer patients [11]. Nesfatin-1 levels in plasma from chronic obstructive pulmonary disease (COPD) patients are correlated with plasma levels of interleukin-6 (IL-6), interleukin-8 (IL-8), and tumor necrosis factor-α (TNF-α), implying that nesfatin-1 acts as a novel inflammatory factor in COPD patients [12, 13]. These findings indicate that nesfatin-1 may be associated with inflammation-related lung diseases. However, the property of nesfatin-1 in regulating the immune system during ALI has not been evaluated. To further explore the mechanism by which nesfatin-1 alleviates ALI, we investigated the protective effect of nesfatin-1 on ALI in an in vivo mice model, as well as its effect on the activation of macrophages in vitro*.*

## Materials and methods

### Animal model establishment

The animal experiments were approved by the Animal Ethics Board of Xiantao First People's Hospital Affiliated with Yangtze University and performed according to the institutional guidelines. The ALI model was established via intratracheal instillation with LPS as described previously [14]. Forty female C57BL/6 mice (20–25 g, Charles River, Wilmington, MA) were randomly separated into four groups (ten mice per group): the vehicle group, intranasal instillation with anequal volume of saline; ALI group, intratracheal instillation with 3 mg/kg LPS 24 h; nesfatin-1 treatment groups, pre-treated with nesfatin-1 **(**1.0, 2.0 μg/kg/day, i.p injection) for 7 days and then induced by LPS (intranasal instillation of 3 mg/kg LPS) 24 h.

After another 24 h, mice were anesthetized, peripheral blood was collected through cardiac puncture. Then the trachea was cannulated with an 18-gauge polypropylene catheter and the lung was lavaged with 1 ml of PBS to obtain BAL fluid. At the end of the experiments, the lung tissues were fixed and collected as previously described [15].

### RT-PCR

RNA from lung samples and cells were isolated and then yielded to RT-PCR analysis with a QuantiTect reverse transcription kit and QuantiFast SYBR Green PCR kit (Qiagen). The relative mRNA levels of IL-6, IL-1β, and TNF-α were calculated after the correction for GAPDH. The following primers were used: IL-6, Forward, 5′-GGCGGATCGGATGTTGTGAT-3′, Reverse, 5′-GGACCCCAGACAATCGGTTG-3′; IL-1β, Forward, 5′-CCTTCCAGGATGAGGACATGA-3′, Reverse, 5′-TGAGT CACAGAGGATGGGCTC-3′; TNF-α, Forward, 5′-TCTTCTCATTCCTGCTTGTG G-3′, Reverse, 5′-CACTTGGTGGTTTGCTACGA-3′; GAPDH, Forward 5′-AATGGATTTGGACGCATTGGT-3′, Reverse 5′-TTTGCACTGGTACGTGTTGAT-3.

### Western blot

Total protein from lung samples and whole-cell lysates were prepared, separated by SDS-PAGE, and then transferred onto membranes. The resulting membranes were incubated with 5% skim milk blocking buffer, incubated with primary antibody against nesfatin-1 (1:1000), p-p38 (1:500), p-c-Jun (1:2000), p-p65 (1:1000),or β-actin (1:5000) (Abcam, Cambridge, MA), followed by incubation with horseradish peroxidase (HRP)- labelled secondary antibody (Abcam). Chemiluminescent system (Bio-Rad, Hercules, CA) was applied for developing the bands on the membranes, which were quantified with Quantity One System (Bio-Rad).

### Hematoxylin–eosin (H&E) staining

Lung tissues fixed in 10% (v/v) formalin were embedded in paraffin and subsequently cut into 5 µm sections. After deparaffinization and rehydration, the sections were collected for H&E staining. The sections were reviewed by a pathologist under a light microscope (Olympus) and the inflammation scores were calculated [16].

### Cells analysis in BAL fluid

The inflammatory cells in cell pellets from BAL fluid were counted with a hemacytometer using an established method [17]. Counting for different inflammatory cells was performed using an Olympus light microscope with standard morphological criteria.

### Enzyme-linked immunosorbent assay (ELISA)

After centrifugation, the mice serum and supernatants were collected and analysed for the contents of IL-6, IL-1β, and TNF-α using the ELISA method with commercially obtained kits (BD Biosciences, Franklin Lakes, NJ). Briefly, an ELISA plate was coated with 100 µL of the antigen dilution and incubated overnight at 4 °C. After blocking with 5% non-fat dry milk for 2 h at room temperature, the plates were washed with PBS twice and diluted antibodies were added for 2 h at room temperature. 100 µL of the substrate solution was added to each well. After sufficient color development, 50–100 µL of stop solution was added to the wells. Finally, an automated spectrophotometric plate reader (PerkinElmer, Waltham, MAA) was applied for detecting the reactions at 450 nm.

### RAW264.7 cells culture

Mouse macrophage cell line, RAW264.7 cells were purchased from ATCC (Manassas, VA) and maintained in 1640 culture medium (Invitrogen, Carlsbad, CA) supplemented with 10% (v/v) FBS and antibiotics. For the LPS treatment group, RAW264.7 cells were treated with 1 μg/ml LPS. For nesfatin-1 intervention groups, cells were pretreated with 50 or 100 nM nesfatin-1 for 1 h and then treated with 1 μg/ml LPS [18].

### Cell counting kit-8 (CCK-8)

Cytotoxicity examination of nesfatin-1 on RAW264.7 cells was performed using the Cell Counting Kit-8 assay after incubation with nesfatin-1 (5, 10, 20, 50, 100, 200, and 400 nM) for 24 h. The pretreated cells were then incubated with CCK-8 solution for 2 h before the optical density was measured at 450 nm.

### Oxidative stress evaluation

Intracellular ROS generation of RAW264.7 cells was measured using the probe H_2_-DCFDA (Invitrogen, Carlsbad, CA). The fluorescence intensity was analyzed using a fluorescence microscope (Carl Zeiss, GmbH, Jena, Germany). SOD enzyme activity was detected using a commercial detection kit (KeyGen Biotech., Nanjing, China).

#### Data analysis

All data were analyzed using SPSS 21.0 software. To determine statistical significance, a one-way analysis of variance was applied for multiple comparisons. Differences with *p* values less than 0.05 were considered significant.

## Results

In order to investigate the potential effects of nesfatin-1 on ALI, we established both an in vivo mice model and in vitro macrophages model. We found that LPS stimulation promotes inflammatory response in the lung tissues of mice, which was significantly inhibited by nesfatin-1. In the in vitro model, the two doses of nesfatin-1 attenuated an inflammatory response and oxidative stress in LPS-challenged macrophages. Importantly, nesfatin-1 alleviated the p38/c-Jun/NF-κB signaling pathway, which plays a critical role in various inflammatory diseases, including ALI.

### The expression of nesfatin-1 was decreased in the ALI mice

**Fig. 1:**
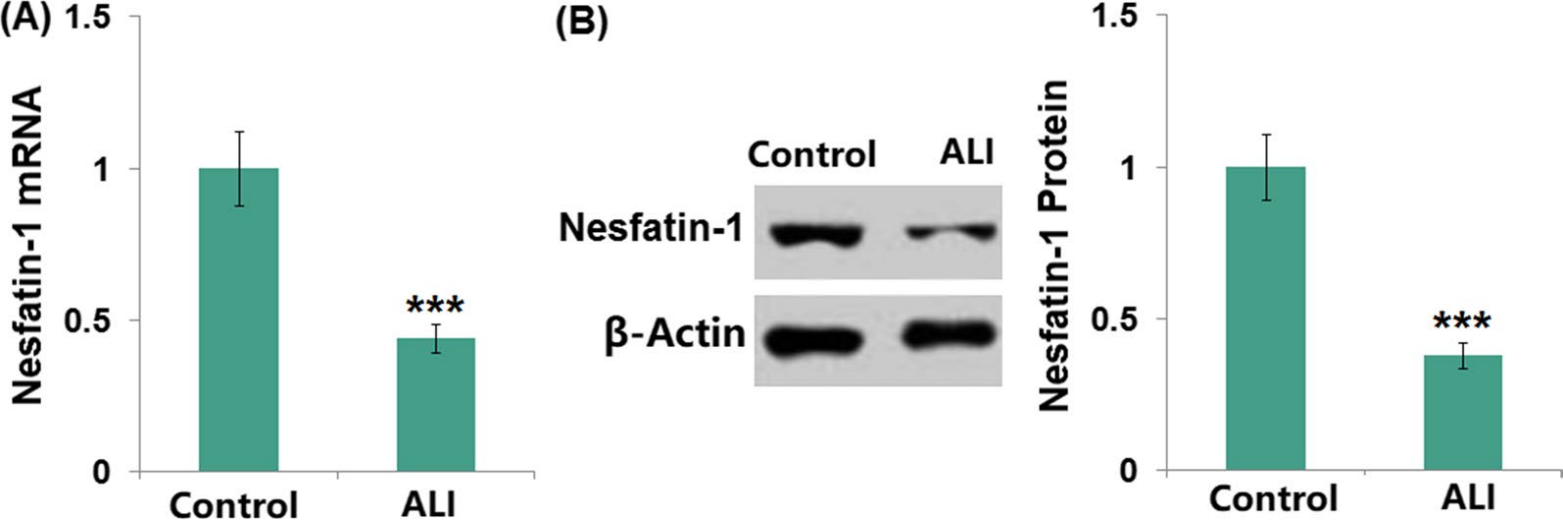
The expression of Nesfatin-1 was decreased in the lung tissue of ALI mice. (**A**). The mRNA level; (**B**). Protein level of Nesfatin-1 was determined in the control and ALI group (***P < 0.0001 vs. vehicle group)

We first compared the expression of nesfatin-1 in the lung tissues from both the control and ALI mice. We found that the mRNA level of nesfatin-1 was significantly reduced by 0.44-fold in ALI mice (Fig. 1A). In line with this, a 0.38-fold reduction of the nesfatin-1 protein level was also observed in the ALI group (Fig. 1B), which suggested a potential role for nesfatin-1 in ALI.

### Nesfatin-1ameliorated the damage in LPS- induced ALI in mice

**Fig. 2:**
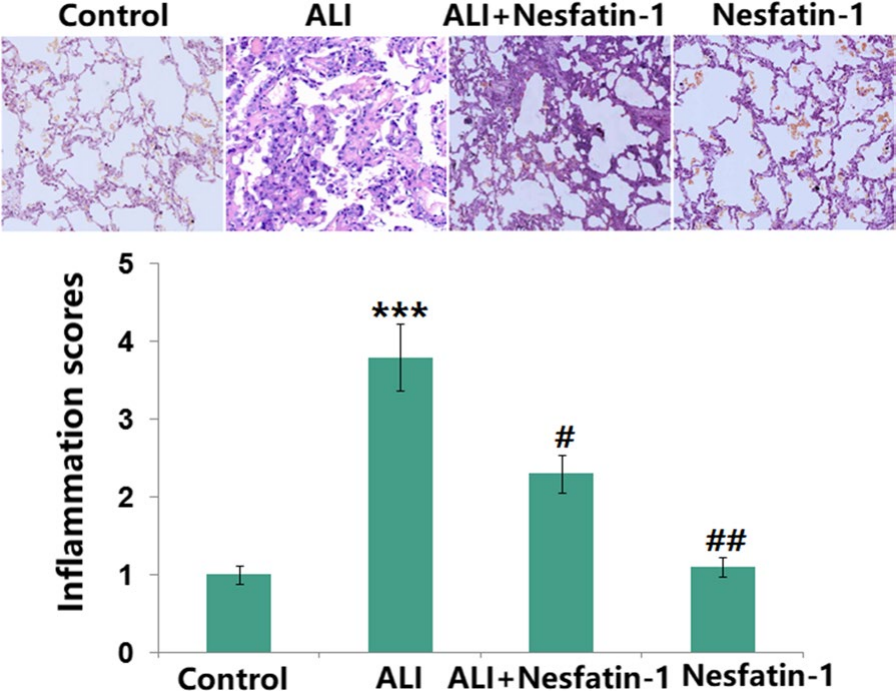
Nesfatin-1 ameliorated the damage in LPS-induced acute lung injury in mice. Lung tissues from each experimental group were processed for histological evaluation. The inflammation scores were calculated (***P < 0.0001 vs. vehicle group; #, ##, *P* < 0.01, 0.001 vs. LPS treatment group)

The ALI mice were administrated with nesfatin-1 (1.0, or 2.0 μg/kg/day) to further evaluate its role. As illustrated in Fig. 2, histological evaluation of lung tissues showed that the inflammation score in ALI mice was increased by 3.8-fold, which was attenuated by nesfatin-1 intervention.

### Nesfatin-1 attenuated LPS-induced inflammatory response

**Fig. 3:**
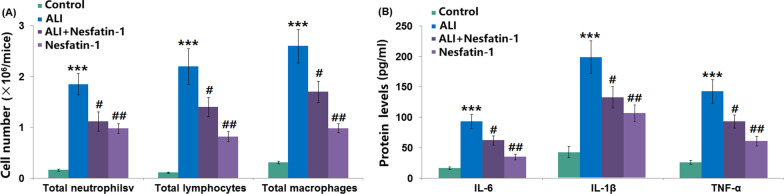
Nesfatin-1 attenuated LPS-induced inflammatory response. (**A**). Total neutrophils, total lymphocytes, and total macrophages were counted; (**B**). IL-6, IL-1β, and TNF-α levels in serum were measured at 24 h after LPS challenge (***P < 0.0001 vs. vehicle group; #, ##, *P* < 0.01, 0.001 vs. LPS treatment group)

Analysis of inflammatory cells in BAL fluid showed that total neutrophils, total lymphocytes, and total macrophages were dramatically increased in ALI mice. However, these increased inflammatory cells were significantly decreased after nesfatin-1 intervention (Fig. 3A). Significant increases in the contents of IL-6 (93.4 ± 11.6 pg/ml), IL-1β (198.7 ± 26.8 pg/ml) and TNF-α (143.1 ± 19.3 pg/ml) in sera from ALI mice were detected, as compared to those in control mice (16.8 ± 2.3, 42.5 ± 4.9, 26.4 ± 3.1 pg/ml). The increased content of these inflammatory cytokines was reduced in nesfatin-1 treatment groups (Fig. 3B).

### Cytoxicity of nesfatin-1 in RAW 264.7 cells

**Fig. 4:**
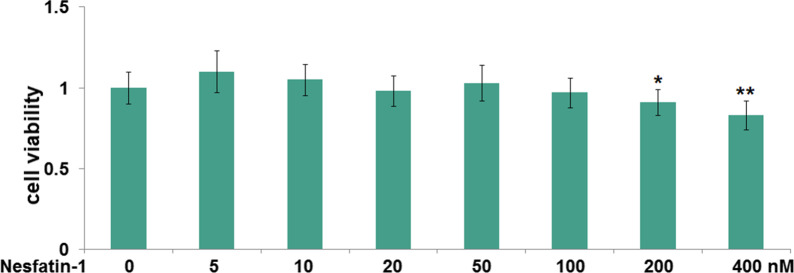
Cytoxicity of nesfatin-1 in RAW 264.7 cells. Cells were treated with Nesfatin-1 (5, 10, 20, 50, 100, 200 and 400 nM) for 24 h. The cell viability was determined with the CCK-8 assay (*, **P < 0.01, 0.001 vs. vehicle group)

Figure 4 shows the cytotoxicity of nesfatin-1 on RAW 264.7 cells, which indicated that cell viability was significantly reduced in 200 or 400 nM nesfatin-1-treated RAW 264.7 cells with 0.91-and 0.83-fold changes, respectively. While at the concentrations of 5, 10, 20, 50, and 100 nM, cell viability was not affected.

### Nesfatin-1 attenuated the generation and release of proinflammatory cytokines in response to LPS stimulation

**Fig. 5:**
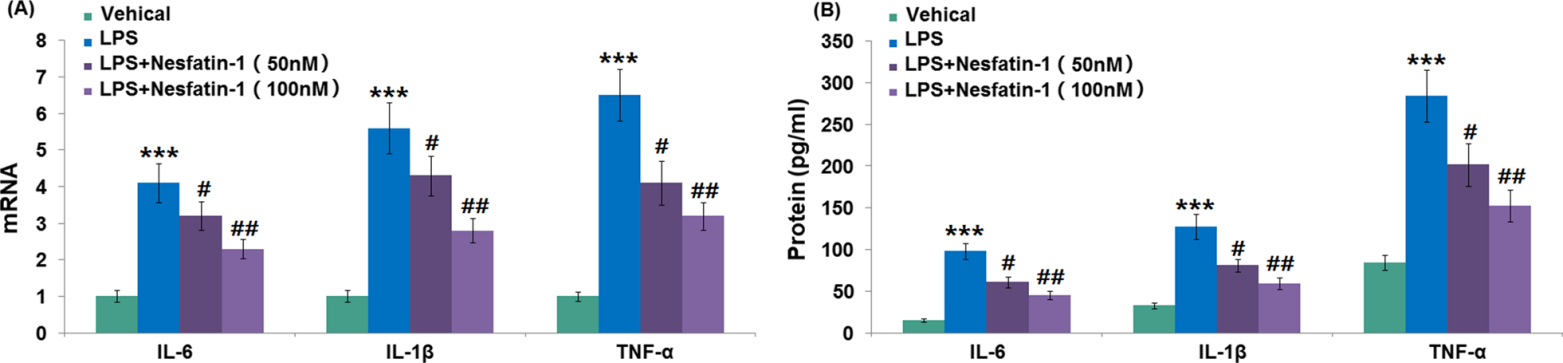
Nesfatin-1 attenuated inflammatory response in LPS-stimulated RAW 264.7 cells. Cells were treated with LPS (1 μg/mL) in the presence and absence of Nesfatin-1 (50, 100 nM). (**A**) The mRNA levels of IL-6, IL-1β, and TNF-α; (**B**) Protein levelsof IL-6, IL-1β and TNF-α (***P < 0.0001 vs. vehicle group; #, ##, *P* < 0.01, 0.001 vs. LPS treatment group)

In Fig. 5A, RT-PCR results reveal that 1 μg/ml LPS dramatically elevated the mRNA levels of IL-6 (4.1 ± 0.53), IL-1β (5.6 ± 0.69), and TNF-α (6.5 ± 0.71) in RAW 264.7 cells relative to control cells. Pre-intervention with 50 or 100 nM nesfatin-1 suppressed the increases in IL-6, IL-1β, and TNF-α mRNA levels. Meanwhile, the elevated protein levels of IL-6 (98.6 ± 9.6 vs. 15.7 ± 1.7 pg/ml), IL-1β (33.4 ± 3.6 vs. 127.8 ± 14.9 pg/ml), and TNF-α (84.9 ± 8.7 vs. 284.3 ± 31.2 pg/ml) in 1 μg/ml LPS-treated RAW 264.7 cells were significantly decreased after 50 or 100 nM nesfatin-1 treatment (Fig. 5B).

### Nesfatin-1 inhibited oxidative stress in LPS-induced RAW 264.7 cells

**Fig. 6:**
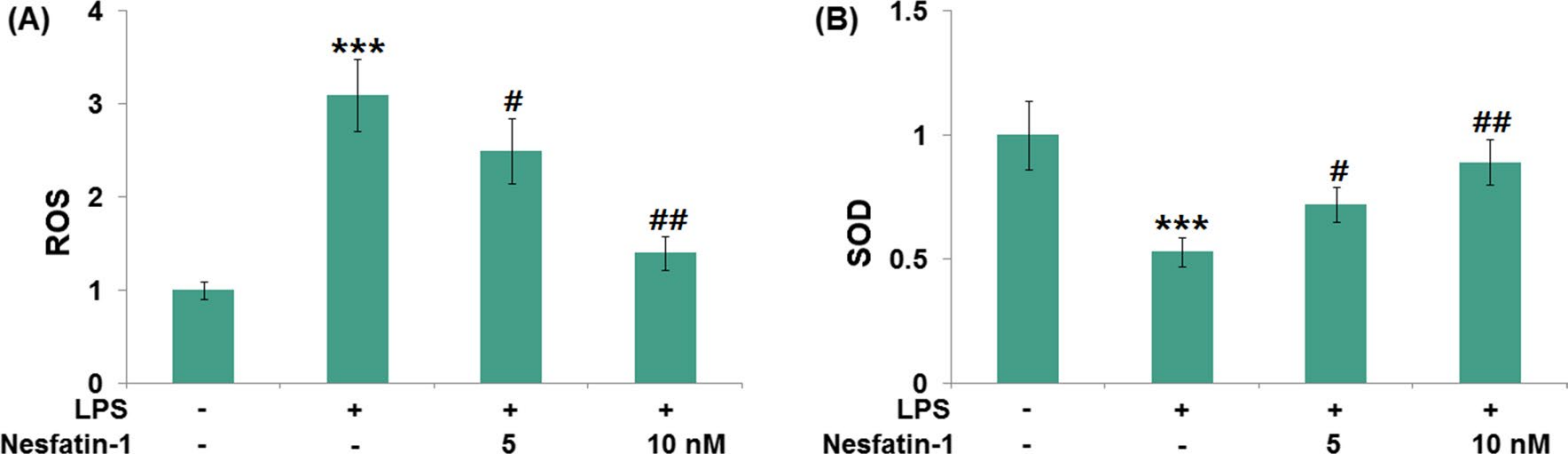
Nesfatin-1 alleviated oxidative stress in LPS-stimulated RAW 264.7 cells. Cells were treated with LPS (1 μg/mL) in the presence and absence of Nesfatin-1 (50, 100 nM). (**A**) ROS level was detected using an intracellular fluorophore (DCFH-DA) assay; (**B**) Activity of SOD was detected using SOD activity assay kit (***P < 0.0001 vs. vehicle group; #, ##, *P* < 0.01, 0.001 vs. LPS treatment group)

LPS is well known as an important risk factor in oxidative injury during ALI. Therefore, LPS is widely used to stimulate macrophages to establish oxidative stress models [19]. As shown in Fig. 6A, ROS production was increased rapidly by 3.1-fold in LPS-induced RAW 264.7 cells with higher fluorescence. However, the increased ROS production was effectively suppressed by nesfatin-1 (50 or 100 nM) treatment. Also, we found that nesfatin-1 (50 or 100 nM) treatment significantly improved the decreased SOD activity (0.53-fold) in LPS-induced RAW 264.7 cells (Fig. 6B).

### Nesfatin-1 inhibited the activation of the p38 MAPK/c-Jun pathway

**Fig. 7:**
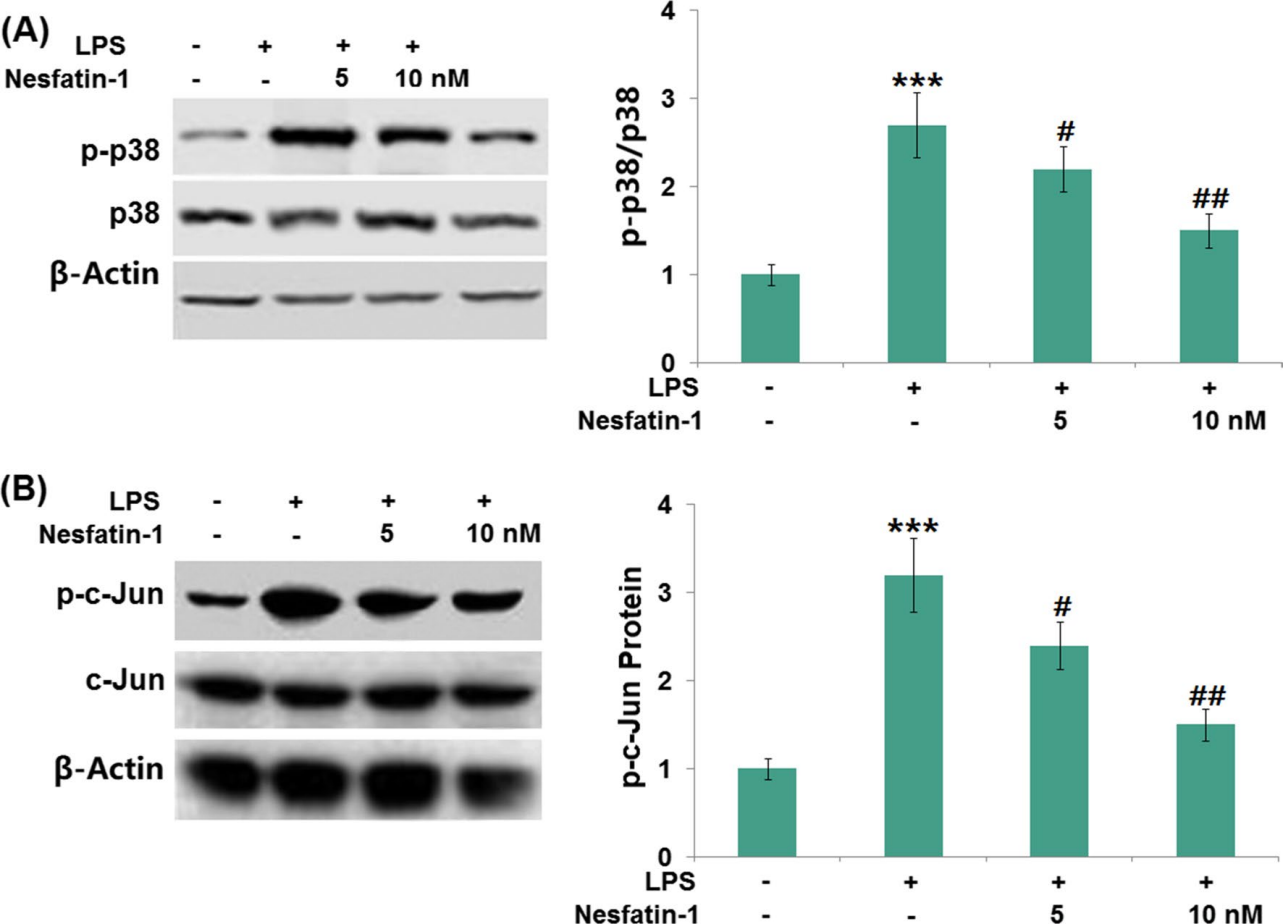
Nesfatin-1 inhibited the phosphorylation of p-p38 and c-jun in LPS-stimulated RAW 264.7 cells. Cells were treated with LPS (1 μg/mL) in the presence and absence of Nesfatin-1 (50, 100 nM). (**A**). Levels of phosphorylated p38; (**B**) Levels of phosphorylated c-Jun (***P < 0.0001 vs. vehicle group; #, ##, *P* < 0.01, 0.001 vs. LPS treatment group)

As shown in Fig. 7A–B, the phosphorylation of p38 and that of c-Jun in RAW 264.7 cells were induced by LPS (1 μg/ml), evidenced by the increased expression levels of p-p38 (2.7-fold) and p-c-Jun (3.2-fold). The inductive effects of LPS (1 μg/ml) on p38 and c-Jun phosphorylation were prevented by nesfatin-1 (50 or 100 nM) treatment.

### Nesfatin-1 inhibited the activation of the NF-κB pathway

**Fig. 8:**
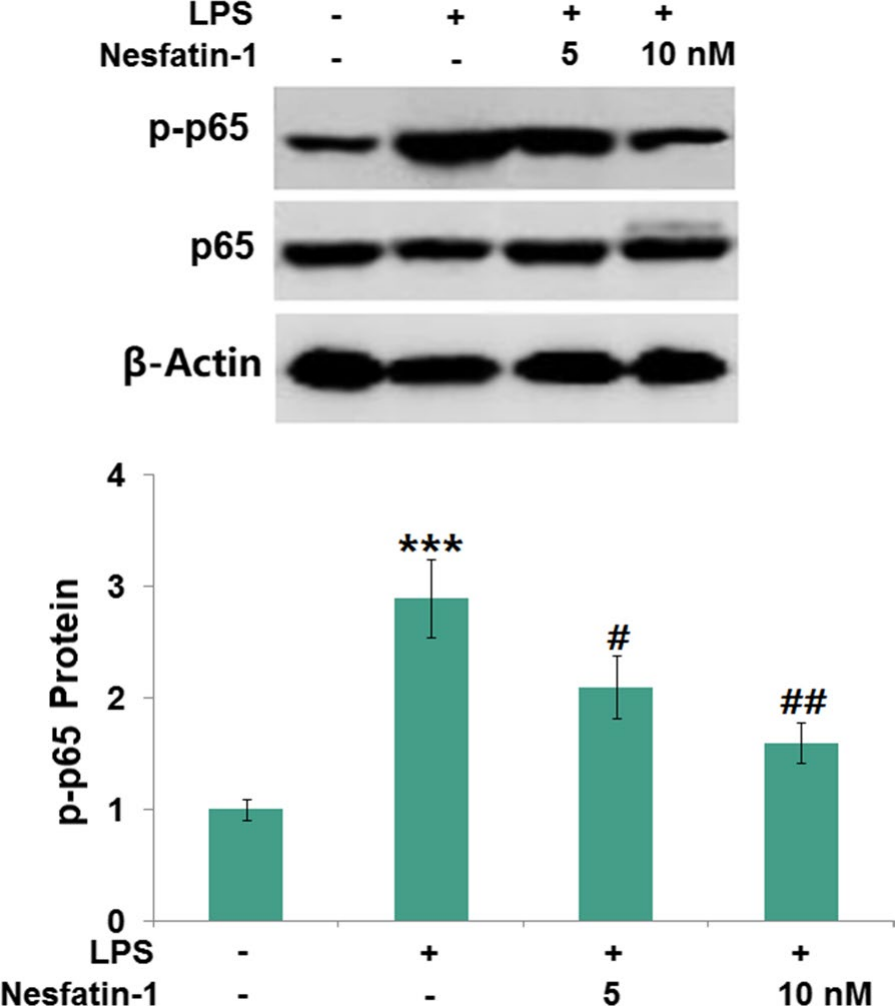
Nesfatin-1 inhibited the activation of NF-κB in LPS-stimulated RAW 264.7 cells. Cells were treated with LPS (1 μg/mL) in the presence and absence of Nesfatin-1 (50, 100 nM). The levels of p-p65 were measured (***P < 0.0001 vs. vehicle group; #, ##, *P* < 0.01, 0.001 vs. LPS treatment group)

Next, we analyzed the phosphorylation of p65 via Western blot, which is presented in Fig. 8. We could find that the levels of p-p65 were markedly increased by 2.9-fold in cells treated with LPS (1 μg/ml). The change in p-p65 expression was abolished after treatment with nesfatin-1 (50 or 100 nM).

## Discussion

Nesfatin-1 is known to be involved in inflammatory responses under various pathological conditions. Treatment with nesfatin-1 protects human aortic endothelial cells (HAECs) from free fatty acids (FFAs)-induced inflammatory injury with decreased release of lactate dehydrogenase (LDH) and generation of inflammatory factors via the Gfi1/NF-κB signaling pathway. Nesfatin-1 ameliorates osteoarthritis in a rat model and suppresses cartilage matrix destruction, inflammation, and apoptosis in IL-1β-induced chondrocytes. Nesfatin-1 attenuates acute myocardial infarction (MI) in a rat model by regulating reperfusion-caused oxidative and inflammatory injury. Nesfatin-1 exerts a protective effect on retinal epithelial cells against high glucose-induced inflammatory injury [20]. These findings indicate that nesfatin-1 is involved in the development of inflammation-related diseases.

We explored the role of nesfatin-1 in ALI, which showed that nesfatin-1 expression was downregulated in the lung tissues in ALI mice. Nesfatin-1 treatment ameliorated the inflammatory response and lung tissue damage in LPS-induced ALI in mice. Consistent with our results, recent research [10] reported the potential effect of nesfatin-1 against LPS- induced ALI. They measured the expressions of IL-1β and TNF-α in LPS- challenged epithelial cells. They found that treatment with nesfatin-1 significantly attenuated the secretion of these two proinflammatory cytokines, demonstrating the inhibitory effect of nesfatin-1 on LPS- induced inflammatory response. Furthermore, they also found that nesfatin-1 suppressed oxidative stress by reducing the generation of ROS and production of malondialdehyde (MDA), and rescuing the production of SOD and Glutathione Peroxidase (GSH-Px), supporting the conclusion from our findings. Hui et al. [21] reported that inflammatory responses including inflammatory cytokine expression and adherent neutrophils accumulation are higher in nesfatin-1-knockout mice than in wild-type mice, implying that loss of nesfatin-1 increases LPS- induced ALI in mice. Our results together with previous studies show that nesfatin-1 exerts a protective effect against ALI. However, the underlying mechanisms must be fully understood.

The underlying immunological mechanism of ALI is related to the uncontrolled excessive lung inflammation, which accounts for the high mortality rate [22]. Macrophages are heterogeneous cell components and serve a key role in the pathogenesis of ALI [23]. Following exposure to pulmonary toxicants, an accumulation of the M1 macrophages (proinflammatory/cytotoxic) is observed at sites of tissue injury, followed by the appearance of M2 macrophages (anti-inflammatory/wound repair) [24]. Thus, the balance between M1 and M2 subpopulations is crucial for the outcome of pathogenic responses to toxicants. Overactivation of either the M1 or M2 subpopulation may serve as the pathogenesis of tissue injury [25]. It is thought that M1 macrophages are activated in response to a range of molecules, such as interferon (IFN)-γ, or in conjunction with TLR4 agonists or other cytokines. LPS is a ligand of TLR4 and develops the M1 macrophages, which generate and release diverse proinflammatory cytokines, proteolytic enzymes, bioactive lipids, cytotoxic RNS, and ROS [26]. Exaggerated activation of M1 macrophages contributes to the persistent inflammation and development of ALI. These evidences indicate that excessively accumulating M1 macrophages play a role in ALI. Here, we investigated whether nesfatin-1 could regulate the LPS-mediated activation of M1 macrophages. In this study, LPS treatment converted macrophages to the M1 phenotype, which exerts inflammatory properties. ELISA revealed a remarkable increase in the expression of proinflammatory cytokines including IL-6, IL-1β, and TNF-α in M1 macrophages. However, nesfatin-1 attenuated the generation and release of proinflammatory cytokines in RAW 264.7 cells in response to LPS induction. Nesfatin-1 also inhibited ROS production and improved SOD activity in LPS- induced RAW 264.7 cells.

In monocytes, LPS stimulation induces activation of a broad range of signaling pathways and transcription factors [27]. Specifically, LPS stimulation binds to LPS-binding protein (LBP) to form a complex, which is then transferred to CD14 locatedon the cell surface [28, 29]. Subsequently, LPS interacts with TLR4 and the accessory protein MD-2, which leads to the activation of intracellular kinases [30, 31]. Among these kinases, MAPK including the ERK, JNK, and p38 pathways, as well as the IKK pathways are important for the activation of various transcription factors, such as c-Jun, c-Fos, ATF-1/2, CREB, and p65 [27]. We found that p38 MAPK/c-Jun and IKK/NF-κB pathways were inhibited by nesfatin-1, as shown by the decreased expression levels of p-p38, p-c-Fos, and p-p65. Recently, Wang ZZ et al*.* reported that Nesfatin-1 reduces the expression of high-mobility group protein B1 (HMGB1) in LPS- stimulated BEAS-2B cells, and inhibits activation of the p38MAPK/NF-κB pathways. However, overexpression of HMGB1 attenuated the protective benefits of nesfatin-1 on ALI [10]. Moreover, Sun H et al. demonstrated that the anti-inflammatory and antioxidant effects of nesfatin-1 on high glucose-treated human retinal epithelial cells were modulated by HMGB1 [20]. Although we did not measure the expression of HMGB1, these findings suggest that the inhibitory effect on the p38MAPK/c-Jun/NF-κB pathways might be mediated by HMGB1.

Collectively, nesfatin-1 alleviated LPS-induced ALI in mice models and alleviated LPS-mediated activation of M1 macrophages. Therefore, the protective property of nesfatin-1 in ALI might be attributed to regulating inflammatory response through macrophages modulation. The limitation of the current study should be addressed. An important limitation is that the molecular mechanisms whereby Nesfatin-1 ameliorated the inflammatory response and lung tissue damage in LPS-induced ALI in mice are still unknown. It should be noted that the pathological mechanism of ALI is complicated and needs to be elucidated. In addition to macrophages, there are several types of cells involved in the initiation and progression of ALI. Despite numerous studied interventions, there are no effective pharmacological therapies for treating ALI to substantially reduce mortality and improve the patients’ quality of life. Although our in vivo experiments using a LPS-challenged rodent model and in vitro experiments using macrophages shed light on the promising application of Nesfatin-1 in ALI, these results are still preliminary. Further investigations with comprehensive models, even clinical trials, will provide more evidence.

## Data Availability

The data that support the findings of this study are available from the corresponding author upon reasonable request.
